# Combining JAK Inhibitors with Immune Checkpoint Inhibitors: Overcoming Resistance in Cancer Treatment

**DOI:** 10.1002/mco2.70141

**Published:** 2025-03-04

**Authors:** Hai‐Jing Zhong

**Affiliations:** ^1^ State Key Laboratory of Bioactive Molecules and Druggability Assessment International Cooperative Laboratory of Traditional Chinese Medicine Modernization and Innovative Drug Development of Chinese Ministry of Education (MOE) of China College of Pharmacy Jinan University Guangzhou China

1

Balancing acute inflammation and chronic inflammation is challenging in cancer immunotherapy while two recent groundbreaking studies published in *Science* have shown a promising strategy to address this challenge by combining Janus kinase (JAK) inhibitors with anti‐PD‐1 therapy [1, 2].

One of these studies, Zak et al. [1] assessed the efficacy of combining the anti‐PD‐1 antibody nivolumab with ruxolitinib in patients with relapsed or refractory Hodgkin lymphoma. Ruxolitinib, the first JAK inhibitor approved by US Food and Drug Administration, has proven to be clinically useful in treating graft‐versus‐host disease and myeloproliferative neoplasms [3]. The combination therapy in the classical Hodgkin lymphoma (CHL) trial demonstrated striking efficacy, achieving a best overall response rate of 53% (10 out of 19). All enrolled patients had previously failed checkpoint inhibitor therapy, with eligibility criteria specifically requiring patients to have exhibited refractory disease, relapsed disease, or stable disease. Notably, significant clinical responses were observed in these patients.

In another clinical trial focusing on metastatic non‐small cell lung cancer (NSCLC), the combination of anti‐PD‐1 therapy (pembrolizumab) and itacitinib, a selective JAK1 inhibitor, demonstrated remarkable efficacy. This study, conducted by Mathew et al., reported an overall response rate of 67% among the 21 treatment‐naïve metastatic NSCLC patients with tumor PD‐L1 ≥50% [2]. This response rate significantly surpasses historical response rate of 44.8% to pembrolizumab monotherapy in NSCLC.

Immune checkpoint inhibitors (ICIs) are widely used as standard treatments for various cancer types; however, their overall response rate remains low across the broader patient population [4]. These two studies in NSCLC and Hodgkin lymphoma show how well the combination works to enhance ICI efficacy and overcome resistance mechanisms (Figure 1).

**FIGURE 1:**
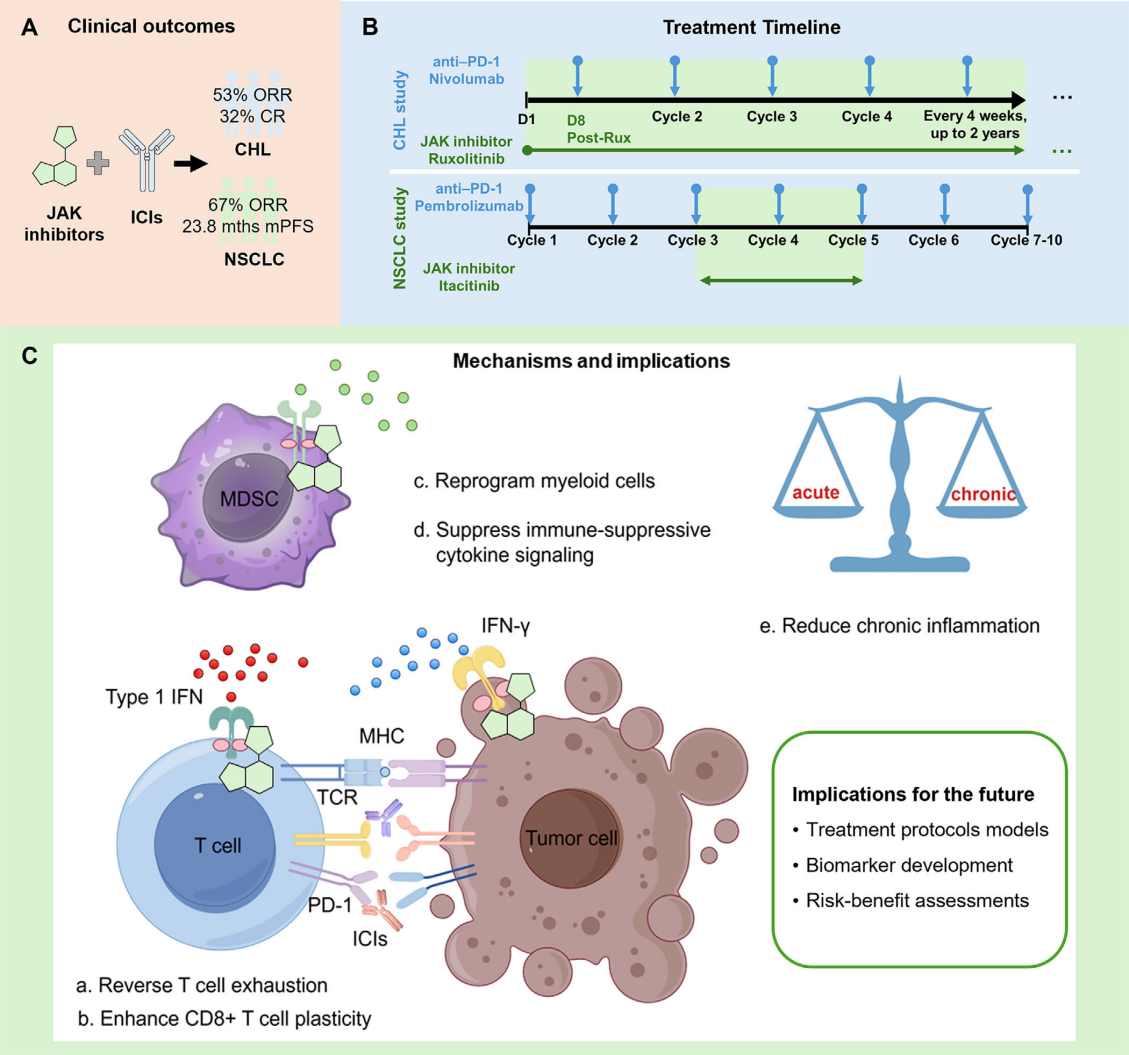
In patients with NSCLC or CHL, immune checkpoint blockade is used in combination with JAK inhibition. (A) Overview of the clinical outcomes observed in the NSCLC and CHL studies. ORR, overall response rate; CR, complete response; mPFS, median progression‐free survival. (B) Detailed timelines of the treatment protocols used in these two clinical trials. (C) Exploration of the possible mechanisms by which JAK inhibitors enhance immune responses against tumors, along with their clinical implications (mechanism illustrated using Figdraw). IFN, interferon; MDSC, myeloid derived suppressor cell; MHC, major histocompatibility complex class; TCR, T cell receptor; PD‐1, programmed cell death protein 1; ICIs, immune checkpoint inhibitors.

JAK inhibitors help address the mixed effects of interferon (IFN) signaling, which can both stimulate and suppress antitumor immunity. While IFNs are important for antiviral and antitumor activity, persistent IFN signaling can lead to immunosuppression and resistance to ICIs therapy. This was demonstrated in the NSCLC study, where patients who responded to JAK inhibitor itacitinib addition showed decreased inflammatory signaling and reduced IFN‐stimulated gene expression. By carefully timing JAK inhibition, the treatment can block the immunosuppressive effects of chronic inflammation while preserving beneficial acute inflammatory responses that support antitumor immunity. In the Hodgkin lymphoma study, the combination of ruxolitinib and checkpoint inhibitors demonstrated a unique ability to preserve and even enhance essential T cell functions, including cytokine production and proliferation, while carefully modulating chronic IFN signaling without completely suppressing it. This balanced approach successfully reduced chronic inflammation through myeloid cell reprogramming while simultaneously maintaining beneficial immune responses, as evidenced by preserved T cell numbers and function in both mouse models and Hodgkin lymphoma patients, with therapeutic doses showing no impairment of critical antitumor immunity. Importantly, the reprogramming of myeloid cells observed appears to result from a synergy between JAK inhibitors and ICIs, as similar effects were not seen with JAK inhibitors alone. JAK inhibitors may sensitize immune cells to activation by reducing chronic inflammation and IFN‐driven resistance, but the ICI signal is required for their full activation and functional reprogramming.

The other mechanism centers on enhancing T cell function and rescuing T cell exhaustion. JAK inhibition, when combined with checkpoint blockade, promotes the development of less terminally differentiated T cell phenotypes and increases the proportion of “fate‐flexible” CD8 T cell progenitor‐like populations. In the NSCLC trial, this was particularly evident as responding patients showed increased numbers of fate‐flexible CD8 T cell progenitors after itacitinib addition, leading to greater T cell plasticity and improved therapeutic responses. This leads to more robust and sustained antitumor immune responses, with T cells showing improved cytokine production, enhanced proliferative capacity, and better cytotoxic action against cancer cells. The Hodgkin lymphoma study demonstrated that ruxolitinib addition could enhance T cell survival, proliferation, and function by reducing apoptosis and increasing cytokine production, and reprogramming myeloid cells from an immunosuppressive state to an immunostimulatory state characterized by increased MHC‐II expression and reduced suppressive markers Arg2, S100a8, and S100a9. Enhanced MHC‐II level improves antigen presentation and recognition by CD4+ T cells, which play a critical role in orchestrating immune responses and supporting CD8+ T cell activity. While CD8+ T cells are primarily responsible for direct cytotoxicity against tumors, the activation and help provided by CD4+ T cells are essential for sustaining a robust immune response [5].

The presented findings hold substantial implications for clinical practice and future research, highlighting critical areas that warrant further investigation. First of all, the timing and context of JAK inhibitor administration are critical for optimizing their effects on T cell exhaustion and overall antitumor immunity. According to Zak and colleagues [1], ruxolitinib's enhancement of T cell responses was not solely due to direct effects on T cells, but also involved indirect effects through modulation of myeloid cells. Their findings suggest that complete blockade of JAK1/2 signaling may be counterproductive for antitumor immunity and ICI efficacy. The work by Mathew et al. further highlighted the importance of timing, showing that delayed administration of a JAK inhibitor after initial anti‐PD‐1 treatment could alter proliferating CD8 T cells and improve checkpoint blockade immunotherapy in mice [2]. These findings underscore the potential of JAK inhibitors to pivot T cell differentiation dynamics and enhance the efficacy of cancer immunotherapy when used in appropriate combinations and sequences. Treatment timing can be adjusted to suit the needs of each patient, creating new opportunities for personalized therapy.

Identifying robust biomarkers to predict patient response to ICI and JAK inhibitor combination therapy remains important, though the relationship between inflammation and treatment response is complex. The Hodgkin lymphoma clinical data revealed clear correlations between treatment response and changes in key inflammatory markers. Complete responders to ruxolitinib plus nivolumab combination therapy demonstrated significantly greater reductions in both neutrophil‐to‐lymphocyte ratio (NLR) and monocyte percentages compared with nonresponders. The NLR, which has established prognostic relevance for progression‐free survival across multiple cancer types including Hodgkin lymphoma, showed marked decreases after ruxolitinib treatment but remained stable following nivolumab administration. This suggests ruxolitinib's specific effect on this marker. Additionally, monocyte levels, which negatively correlate with nivolumab response, were substantially reduced in complete responders, with transcriptional analysis revealing a monocyte‐enriched gene cluster that was more strongly downregulated in these patients. This cluster included important myeloid and myeloid‐derived suppressor cell‐related genes like CD163 and S100A8/9, which remained suppressed even after nivolumab treatment, indicating a durable reshaping of the myeloid compartment that may contribute to improved therapeutic outcomes. The NSCLC study revealed that nonresponders had the highest baseline inflammation that was refractory to JAK inhibition, while JAK inhibitor responders demonstrated specific immunological changes after itacitinib treatment, including decreased inflammatory signaling and increased “fate‐flexible” CD8 T cell progenitor‐like populations. This nuanced understanding of how inflammation, immune responses, and treatment outcomes interrelate may inform future biomarker development efforts.

The positive safety profiles are noteworthy in these two clinical trials. Because of their good tolerability, these medicines may be more widely applicable to individuals who would not otherwise qualify because of comorbidities or frailty. Nonetheless, it's imperative to proceed cautiously with this hope, considering the significant safety issues connected to certain JAK inhibitors, such as elevated risks of malignancies and cardiovascular disease [3].

In conclusion, JAK inhibition combined with ICI offers a promising cancer immunotherapy strategy, enhancing efficacy, overcoming resistance, and balancing acute antitumor immunity with chronic inflammation's immunosuppressive effects.

## Author Contributions

Conception and design, manuscript writing, funding acquisition, and final approval of manuscript: Hai‐Jing Zhong.

## Conflicts of Interest

The author declares no conflicts of interest.

## Ethics Statement

The author has nothing to report.

## Data Availability

The author has nothing to report.
